# Mapping the burden prevalence of neural tube defects across Indian regions: a systematic review and meta-analysis

**DOI:** 10.11604/pamj.2025.52.54.48035

**Published:** 2025-10-01

**Authors:** Urmila Sinha, Shiv Kumar Mudgal, Ajay Kumar Patel, Vipin Patidar, Sanjay Kumar

**Affiliations:** 1 Department of Anatomy, All India Institute of Medical Sciences, Deoghar, Jharkhand, India; 2 College of Nursing, All India Institute of Medical Sciences, Deoghar, Jharkhand; 3 Department of Anesthesiology, All India Institute of Medical Sciences, Deoghar, Jharkhand, India

**Keywords:** Burden, Indian region, neural tube defects, prevalence

## Abstract

Neural tube defects (NTDs) are a major public health concern that greatly raises newborn mortality and morbidity rates. The aim of this systematic review and meta-analysis was to determine the overall prevalence of NTDs across various regions of India. A random-effects model was used to analyze 33 observational studies with a total of 963,577 births from five Indian regions (North, South, East, West, and Central) that were obtained from PubMed, Embase, and Google Scholar. The overall pooled prevalence was found to be 5.05 per 1,000 births (95% CI: 2.68-9.49), with high heterogeneity (I^2^=98.9%). The prevalence varied significantly by region: south (6.61 per 1,000), north (6.50), central (4.66), west (4.21), and east (0.81). Urban areas had higher prevalence rates (8.92 per 1,000) than mixed settings (2.64 per 1,000), although these differences between were not statistically significant. The findings highlight the importance of increasing folic acid fortification, improving prenatal care, addressing regional healthcare imbalances, and intra-regional factors like dietary practices.

## Introduction

Neural tube defects (NTDs) are serious birth abnormalities caused by the failure of the neural tube to close properly, usually in the first month of pregnancy, resulting in anencephaly, spina bifida, and encephalocele [1]. According to estimates, NTDs cause about 300,000 cases and 88,000 deaths annually, making them a significant cause of infant mortality, morbidity, and permanent disability worldwide [2]. Even though the prevalence of NTDs in high-income countries (0.5-2 per 1,000 births) has significantly decreased due to advancements in prenatal care and mandatory folic acid fortification but low- and middle-income countries (LMICs) still have disproportionately high rates, indicating differences in access to healthcare, nutritional status, and public health policies [3,4]. Neural tube defects remain a major public health concern in India, where the nationalwide prevalence rate was 9.46 per 1,000 live births [5]. However, this burden was not evenly distributed throughout the country, with notable state-wise differences, as northern states, like Rajasthan, report prevalence rates as high as 61.22 per 1,000 live births, while southern states, like Tamil Nadu, report much lower rates of 2.23 per 1,000 [6].

India‘s diverse geography, which includes a range of social and economic backgrounds, dietary practices, and healthcare facilities, makes a challenging epidemiological environment for NTDs. In Uttar Pradesh, a state in the northern India, the prevalence rate was 7.76 per 1,000 live births [7]. Similarly, in Odisha, the eastern Indian state, a prevalence of NTDs was 15.96 per 1,000 live births [8]. However, maybe due to improved access to healthcare and successful maternal nutrition programs, southern states like Tamil Nadu and Kerala record lower prevalence rates (2.33 and 2.80 per 1,000) respectively [9,10]. Even these disparities are noticeable within states, as in Maharashtra, urban areas record a prevalence of 7.18 per 1,000 live births, whereas rural areas almost double the rates [11]. Regional gaps are nonetheless worsened by persistent differences in rural-urban healthcare delivery and uneven implementation of fortification policies, even in spite of national initiatives like the Pradhan Mantri Surakshit Matriva Abhiyan (PMSMA), which aims to improve maternal health [12,13]. Countrywide common modifiable risk factors remain in existence, including maternal folate insufficiency, obesity, diabetes, and exposure to teratogenic chemicals, etc. [14,15]. High-income regions confront issues with fair access to prenatal care and genetic counseling, whereas Low-income regions suffer with inadequate healthcare infrastructure [16]. So, it is essential to map the burden of NTDs at the regional level in order to pinpoint context-specific factors and improve preventative measures.

To our best knowledge, this study addresses a significant gap in the literature by being the first thorough meta-analysis to assess the prevalence of neural tube defects (NTDs) across Indian regions as previous studies have only concentrated on specific states or areas, and none have compiled region-specific prevalence rates in a systematic manner to facilitate cross-regional comparisons. This kind of approach is essential for expanding knowledge of NTDs nationalwide, addressing the root causes of NTDs-related disparities, adjusting prevention tactics to regional circumstances, improving resource allocation, and guiding the development of policies that address disparities in maternal and child health outcomes. The objectives of this systematic review and meta-analysis are to: i) estimate the pooled prevalence of NTDs across different Indian regions.

## Methods

**Study design and registration:** the systematic review and meta-analysis were preemptively registered with PROSPERO (CRD42025644538) and followed the Preferred Reporting Items for Systematic Reviews and Meta-Analyses (PRISMA) guidelines [17].

**Search strategy:** several databases, including PubMed, Embase, and Google Scholar, were thoroughly searched from the beginning of the study (March 2025) until May 2025. The search strategy included ("Neural Tube Defects"[Mesh] OR "neural tube defects" OR "anencephaly" OR "encephalocele" OR "spina bifida" OR "meningocele" OR "myelomeningocele") AND ("prevalence" OR "incidence" OR "epidemiology"). The detailed search strategy is given in Annex 1. The reference lists from the included studies and grey literature were also carefully examined.

**Eligibility criteria:** the inclusion criteria were observational studies, including cross-sectional, cohort, prospective and case-control designs, that documented the prevalence or risk factors for NTDs in live births, stillbirths, or terminated pregnancies. Whereas, research without the denominator data required to calculate prevalence, case reports, and review articles were excluded.

**Study selection and data extraction:** the eligibility and data extraction process was done by two independent reviewers, who first screened abstracts and titles before assessing entire texts. Any disagreements were resolved by consensus or through consultation with a third reviewer. Number of NTDs cases, prevalence rates (measured per 1000 births), number of live and still births, and study parameters (author, year, region, state, area of residence, sample size) were carefully extracted into a standardized template.

**Quality assessment:** the Joanna Briggs Institute (JBI) used the JBI Critical Appraisal Checklist for Prevalence Studies to evaluate the studies’ quality. Nine items in this assessment test include "yes," "no" or "unclear" response possibilities. One point was awarded for each item that complied, whereas 0 points were awarded for confusing or non-compliant responses. The methodological quality was evaluated using the overall score, where 0-3 represented low quality, 4-6 represented moderate quality, and 7-9 represented good quality [18].

**Statistical analysis:** the pooled prevalence of NTDs across the Indian regions (North, South, East, West, and Central) was calculated using a random-effects model that adjusts for heterogeneity with a fixed-effects model was used, and results were reported as 95% CI and per 1,000 births. Subgroup analyses were conducted according to area of residence. Cochran‘s Q-test and I^2^ statistics were used to evaluate heterogeneity, whereas Egger‘s regression test and funnel plots were used to determine the publication bias. All analysis was performed in R software version 4.2 using the “metaprop” command.

**Ethical considerations:** the analysis of publicly available data served as the foundation for this research; hence, ethical approval was deemed unnecessary.

## Results

**Figure 1:**
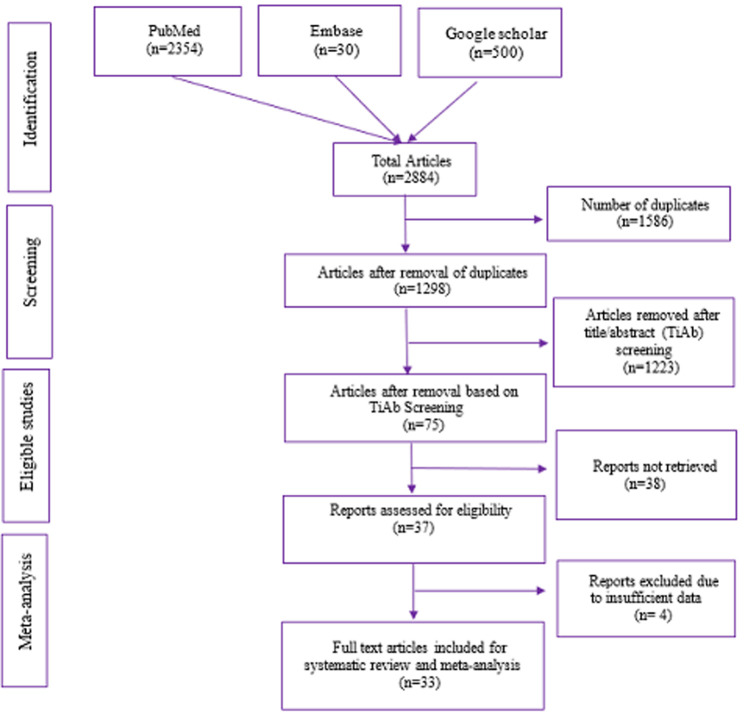
prisma flow chart

**Search results and study characteristics:** our search in the databases of PubMed, Embase and Google Scholar yielded 2884 academic publications by using the keywords specified in our methodology and limiting our search parameters to only include free full-text articles published in English. After removing 1586 duplicates from the combined list of articles, we examined 1298 items in detail for the title-abstract screening. Four studies were excluded as they were not appropriate for pooled analysis because of missing prevalence estimates and insufficient denominator data. The PRISMA flow diagram shown in Figure 1 provides all the specifics related to the screening procedure. In the end, 33 studies [19-49] were included in the meta-analytical evaluation, with a total of 963,577 samples shown in Annex 1. Twelve of these studies are from the South region, twelve are from the North region, and three are each from the Central, West, and East regions of India. The prevalence variation was very high (0.28-744.68/1000 births), with a sample size ranging from 47 to 248,678 births.

**Figure 2:**
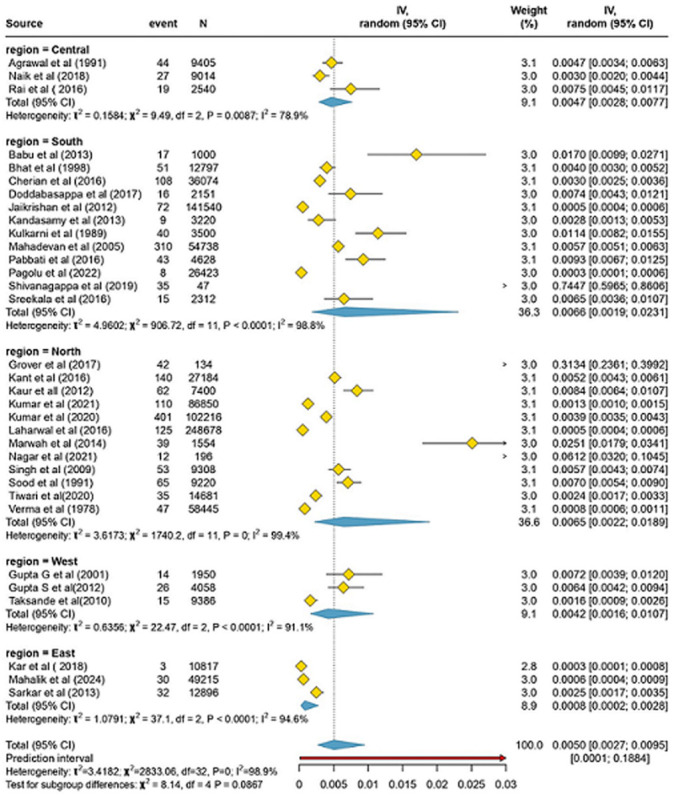
regional prevalence of neural tube defects in India

**Regional prevalence of neural tube defects:** the overall pooled prevalence of neural tube defects (NTDs) was determined using the random-effects model, which was 5.05 per 1000 births (95% CI: 2.68-9.49) over a total of 963,577 births. This showed significant heterogeneity (I^2^=98.9%, P<.001) and a broad prediction interval (0.11-188.43 per 1000), indicating significant variability, as illustrated in Figure 2. Regional analyses revealed different prevalence estimates as Central (4.66 per 1000), South (6.61 per 1000), North (6.50 per 1000), West (4.21 per 1000), and East (0.81 per 1000), all of which had high heterogeneity (I^2^=78.9%-99.4%). The lack of statistically significant results from comparisons between different regions (X4^2^=8.14, P=0.09) suggests that regional differences may not be clinically significant.

**Figure 3:**
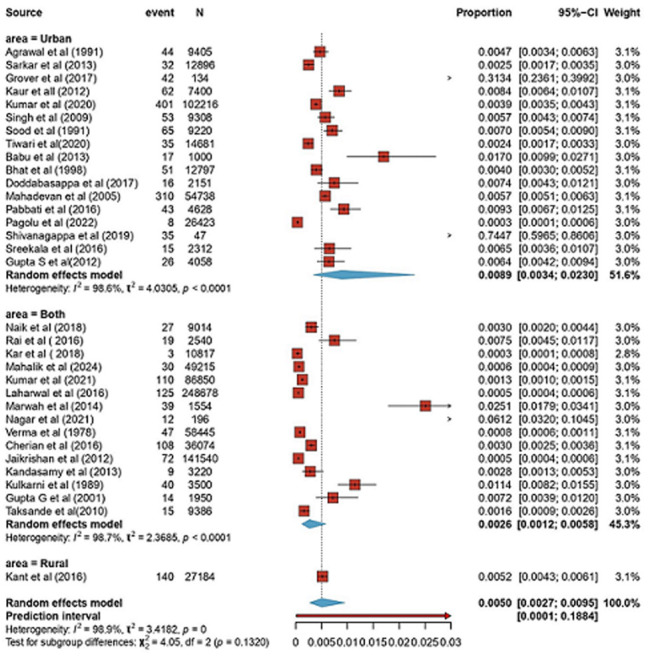
area of residence wise subgroup analysis regarding prevalence of neural tube defects in India

**Subgroup analysis:** the area of residence wise subgroup analysis revealed prevalence rates of 8.92 per 1000 in urban areas (95% CI: 3.44-22.96; I^2^=98.6%), 2.64 per 1000 in both urban and rural areas (95% CI: 1.20-5.78; I^2^=98.7%), and 5.05 per 1000 in rural areas (95% CI: 2.68-9.49; single study), as illustrated in Figure 3. Although the estimates from individual studies differed significantly, the subgroup variances across the different localities were not statistically significant (χ^2^=4.05, P=.13), suggesting that there was not much variation.

**Figure 4:**
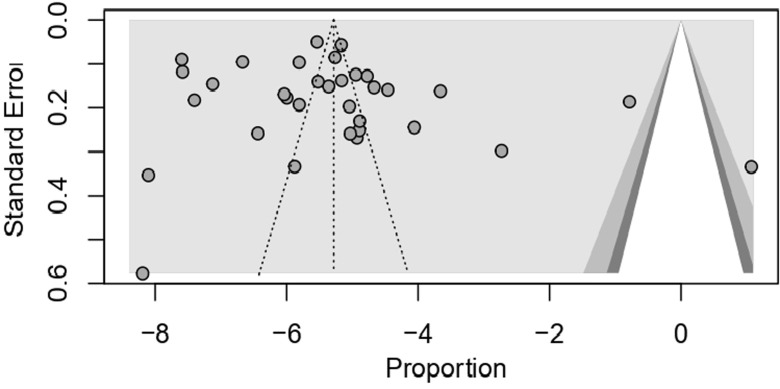
funnel plot showing publication bias

**Sensitivity analysis and publication bias:** we conducted a sensitivity analysis, as the level of heterogeneity among the included studies was quite high (98.9%). A sensitivity analysis using the leave-one-out methodology was performed to identify the studies that significantly affected the total effect size. A nonsignificant bias estimate of 3.33 (SE= 3.26) and no significant publication bias were found using the Egger test for funnel plot asymmetry (t= 1.02, p=0.314) shown in Figure 4 The analysis, which used inverse variance weighting and standard error as the predictor, indicating symmetry in the effect size distribution and no systematic bias in the meta-analysis, even if there was significant residual heterogeneity (τ 2 = 88.41).

**Risk of bias:** the included studies JBI values for risk of bias varied from 3 to 9. Two reports have been classified as low quality, five studies as moderate quality, and twenty-six of the thirty-three studies as high quality. Individual study related information is presented in Annex 1.

## Discussion

This systematic review and meta-analysis examine the regional prevalence of neural tube defects (NTDs) across India. The previous national estimates are consistent with the overall pooled prevalence of this meta-analysis (5.05 per 1,000), but it also explains notable geographical variations ranging from 0.81 per 1,000 in the East to 6.61 per 1,000 in the South that might be impacted by a variety of factors like socioeconomic status, dietary factors, area of residence, and access to healthcare services [5,50]. Prevalence rates of NTDs were greater in the North and South Indian regions, which may be related to their unequal access to healthcare, socioeconomic inequality, and folate insufficiency, but subgroup differences between the regions were not statistically significant indicating that regional differences might not have as much of an influence as previously thought, perhaps this could be because of intra-regional variations like gaps in awareness, accessibility, and compliance [5]. Although there is lack of statistical significance in regional differences, the descriptive variance is still significant, with the East having a prevalence that is almost eight times higher than the South. Policymakers should evaluate these discrepancies as priority signals for focused actions because they are of significant public health concern, especially in the East and North, where prevalence is usually higher.

The area of residence-wise subgroup analyses showed that the prevalence of NTDs was more common in urban regions despite better diagnostic capacities than in mixed or rural areas, which may be a sign of underdiagnoses in places with limited resources [24,40]. Although these differences were descriptively evident, they were not statistically significant. This might be because of unmeasured confounding variables like environmental exposures or intra-regional disparities in dietary practices or healthcare quality [51]. The pooled prevalence needs to be considered carefully due to the extremely high heterogeneity found in this study, even in spite of non-significant publication bias. Geographic and socioeconomic diversity across Indian regions that affect maternal nutrition and folic acid supplementation, variations in study design (hospital-based versus community-based surveillance), diagnostic criteria, case ascertainment techniques, and sample size variability with smaller studies having disproportionate effects are likely the causes of the high heterogeneity. One included study found an exceptionally high prevalence due to both a limited sample size and selective sampling of mothers admitted with fetal congenital abnormalities. While the data were correct as reported, this outlier most likely inflated the total pooled estimate, contributing to the significant heterogeneity [44]. The result revealed a very wide prediction interval, which is indicative of the uncertainty surrounding the actual prevalence statistics. This broad range indicates significant variety in the actual consequences that may occur in various populations. Therefore, the pooled estimate is not a precise prevalence value that applies to all contexts or regions, it should be considered a general indicator of the national burden.

The high national prevalence calls for the expansion of folic acid fortification and prenatal care programs, especially in high-burden regions and states of India, even if regional variations were not statistically significant. It is essential to implement interventions that address local risk factors (such as consanguinity) and enhance access to healthcare in rural areas. In order to improve preventative methods, future research should give priority to longitudinal designs, uniform reporting, and the integration of genetic and environmental risk assessments. The study‘s limitations include an excessive dependence on hospital-based data, which may disproportionately reflect severe instances. The findings’ generalizability is further limited by the variation in study design, limited research in several regions, and small sample size, underscoring the necessity of rigorous methodological frameworks in future research projects.

## Conclusion

The meta-analysis and systematic review show that the burden of NTDs is significant and varies throughout India. A multimodal strategy addressing NTDs in India necessitates coordinated efforts to improve data standards, emphasize maternal nutrition, other dietary interventions, expand maternal health services, and modify regional health policies in order to meet international congenital anomaly prevention criteria.

***What is known about this topic***

- *Neural tube defects show significant geographical disparities in India, which is also a significant contributor to neonatal mortality and morbidity globally;*
- *National prevalence estimates exist but lack regional granularity*

***What this study adds***

- *First meta-analysis quantifying neural tube defects prevalence across Indian regions, which reveals unexpected urban-rural differentials;*
- *It provides region-specific burden estimates to guide targeted interventions.*

## Annex

**Annex 1**: supplementary materials (PDF 407KB)
